# Neurobehavioral concomitants of alcohol use in older healthy adults

**DOI:** 10.3389/fnhum.2025.1693330

**Published:** 2025-11-19

**Authors:** Sara Jo Nixon, Samuel A. Torres, Jeff Boissoneault, Christian C. Garcia, Ben Lewis

**Affiliations:** 1 Department of Psychiatry, University of Florida, Gainesville, FL, United States; 2 Department of Psychology, University of Florida, Gainesville, FL, United States; 3 Department of Neuroscience, University of Florida, Gainesville, FL, United States; 4 UF Center for Addiction Research and Education, Gainesville, FL, United States; 5 Department of Anesthesiology, University of Minnesota, Minneapolis, MN, United States; 6 Department of Psychology, University of Cincinnati, Cincinnati, OH, United States

**Keywords:** aging, alcohol, neurophysiology, neurobehavior, working memory

## Abstract

Few laboratory studies permitting granular analyses of alcohol use on neurobehavioral processes in older adults have been reported. This study, reporting baseline data from an on-going longitudinal project, seeks to address this gap. Toward that end, working memory (WM) processes were targeted using the continuous recognition version of the Mnemonic Similarity Task (MST). Healthy male and female drinkers aged 65–80 years completed self-report measures of substance use, negative affect and demographics prior to testing. Drinking patterns were quantified on the basis of typical standard drinks/day (D/D). Behavioral data were obtained in a two-button forced choice paradigm. Neurophysiological data were obtained for each stimulus presentation with analyses focusing on a mid-frontal negative shift occurring ∼ 300–500 ms post stimulus (FN400) and a posterior positive shift occurring ∼ 550–800 ms after stimulus presentation (LPC). To constrain the models, for the behavioral analyses correlations between D/D, measures of negative affect, stimulus condition (“new,” “identical,” or “similar”) and performance were conducted. They indicated that only accuracy in labeling “new” items was related to D/D. Subsequent least squares regression revealed that D/D was inversely related to accuracy for new items. In a sensitivity analysis removing THC users, the D/D effect was retained. Correlations incorporating mean amplitudes for the FN400 and LPC failed to reveal identifiable patterns. Consequently, separate mixed models (e.g., stimulus condition) for FN400 and LPC were conducted. D/D was not predictive of the FN400 for any stimulus condition. It was negatively related to the LPC mean amplitude. In *post-hoc* analyses, the effect was most notable for “new” stimuli. After removing THC users, the magnitude and direction of the D/D effect was retained, although the p value fell short of significance. Primary models failed to reveal sex main or interaction effects. However, exploratory *post-hoc* analyses justify their continued study. These data lend preliminary support for the hypothesis that sustained drinking among older adults may negatively impact neurobehavioral processes. They are also consistent with expectations that alcohol effects may be modest and constrained by specific process. Importantly, these outcomes will be expanded through on-going longitudinal study, extending investigation to study of alcohol-related cognitive decline.

## Introduction

Although widely observed, age-related compromise in neurobiobehavioral processes is heterogeneous, varying both within and between individuals. In addition to task difficulty, numerous factors contribute to individual differences in cognitive decline including variation in cognitive reserve, compensatory processes, genes, and lifestyle factors (Aichele et al., 2016; Cabeza et al., 2018; Sabia et al., 2014; Salthouse, 2012; Sebastian et al., 2013; Spreng and Turner, 2019; Yang et al., 2023). Lifestyle factors are modifiable and play significant roles in enhancing the quality of life, particularly in older ages. Long-recognized for playing a role in the quality of life across the lifespan, a lifestyle factor that is receiving increased attention is alcohol use among older adults.

There is a well-established literature demonstrating that chronic heavy alcohol use is accompanied by generalized, mild, neurobiobehavioral compromise persisting beyond detoxification (review Nixon and Lewis, 2020). As observed in studies of cognitive aging, the severity of observed impairment varies as a function of task and participant characteristics. There are, however, replicable patterns of alcohol-related impairment that include compromise across diverse domains including attention, working memory, and executive functions (Nixon and Lewis, 2020; Oscar-Berman and Marinkovic, 2007). Although incomplete, the overlap in alcohol and age-related deficits gives rise to increasing concern that continued heavy alcohol use with increasing age may exacerbate cognitive aging processes. Despite limited age ranges within individual samples (e.g., Nixon et al., 2023) laboratory studies of heavy, detoxified drinkers provide compelling data that heavy use differentially impacts cognitive aging processes (Battista et al., 2025; Oscar-Berman and Marinkovic, 2007; Sullivan and Pfefferbaum, 2019; Zahr, 2024).

Importantly while rates of binge drinking and alcohol use disorder (AUD) have increased in older adults (see Keyes, 2023), the majority of older drinkers do not exhibit these patterns (Breslow et al., 2017). There is a substantial epidemiological literature addressing the neurobehavioral and health consequences of alcohol use in drinkers without AUD (i.e., moderate or social drinkers). In contrast to investigations of chronic heavy use, these studies yield inconsistent conclusions with some reporting cognitive benefit, others no effect and still others concluding that any level of consumption carries risk for neurobiobehavioral compromise (Chosy et al., 2022; Hassing, 2018; Lobo et al., 2010; Moussa et al., 2014; Reas et al., 2016; Topiwala et al., 2017). Critically, there is a scarcity of relevant laboratory studies that would permit more granular interrogation (but see Funk-White et al., 2023).

In sum, epidemiological trends and extant knowledge regarding cognitive aging processes and alcohol-related impairment provide a cogent argument for systematic study of alcohol effects on cognitive impairment in older adults. Yet, little systematic effort has been directed to the issue. The current investigation, representing baseline assessment obtained in the course of a 3 year longitudinal study, is an initial effort to address this gap. Recognizing our inability to account for the plethora of potential covariates, we limited study to men and women between the ages of 65 and 80 who were continuing to regularly consume alcohol and who did not have significant medical or psychiatric conditions. Because they are compromised as a function of both alcohol use and aging, we directed attention to working memory (WM) processes and obtained both behavioral and neurophysiological data during the administration of the continuous recognition variant of the Mnemonic Similarity Task (Stark et al., 2019) Extrapolating from studies of alcohol use disorder (AUD) and aging, we predicted that higher levels of typical drinking would be inversely related to WM behavioral performance and account for significant variability in neurophysiological responses. We interrogated two neurophysiological components purportedly underlying recognition memory, a negative going shift occurring between ∼ 300 – 500 ms at mid-frontal sites (i.e., FN400) and a positive going shift occurring between 500 and 800 ms at parietal sites (i.e., late positive component: LPC) (Kwon et al., 2023; Rugg and Curran, 2007; Yang et al., 2019). The first is presumed to reflect the role of item familiarity in recognition processes while the second reflects processes of recollecting whether the item was presented during the task. We anticipated that one or both might be impacted by the variables of interest and thus explored their vulnerability in the context of empirical questions. Finally, recent reports note a dramatic increase in cannabis use, specifically THC, among older adults (Han and Palamar, 2020; Kepner et al., 2023). Therefore, although participants were not selected on the basis of cannabis use, we leveraged the opportunity to obtain information regarding current THC use. These data, while not of primary interest, provide additional context regarding substance use in older adults.

## Materials and methods

This study was approved by the University of Florida (FWA00005790) Institutional Review Board-01 and was conducted in accordance with local legislation and institutional requirements. The participants provided written informed consent prior to initiating study activities and were compensated for their time.

### Participants

The study sample included 124 (*n* = 70 women) healthy older current drinkers. Consistent with typical U.S. definitions of “older” drinkers, we applied a minimum threshold of age 65. Based on earlier data and community input, the upper threshold was age 80. Participants were recruited through radio and newspaper ads, contact registries, and outreach to community groups. Selection criteria permitted educational levels >10 years. Three individuals with educational levels less than 12 were screened, but failed to meet remaining selection criteria and were not considered further.

### Additional selection criteria

Eligible participants were required to score within the unimpaired range on standard assessments of cognitive status (the Telephone Interview for Cognitive Status (TICS, Brandt et al., 1988) if screened remotely and the Montreal Cognitive Assessment (MoCA, Nasreddine et al., 2005) if screened in person). Individuals with common age-related health conditions such as hypertension, high cholesterol and Type 2 diabetes were included if they reported no changes in their on-going treatment over the last month and, if having Type 2 diabetes were not being treated with insulin. Histories of significant health conditions or events (e.g., major cardiovascular events, epilepsy, brain cancer), current or lifetime significant psychiatric diagnoses (e.g., bipolar disorder, major depressive disorder, psychotic disorders) were exclusionary. Eligibility also required no history of significant alcohol problems. nor personal efforts to reduce or quit drinking and no intention to change their drinking patterns. Participants were excluded if they reported lifetime histories of heavy use of other substances or of meeting criteria for another substance use disorder, with the exception of cigarette smoking. To enhance the representativeness of the sample, nicotine use was not exclusionary. Forty-nine of the participants reported histories of regular smoking. Of these, 39 quit more than 10 years ago, another 3 quit between 3 and 10 years prior and 2 quit more recently, but before study enrollment. Among those continuing to use nicotine, one participant reported smoking ∼ three cigarettes/month and two were regular smokers.

### Alcohol use

The quantity frequency index (QFI, modified Cahalan et al., 1969) was derived to provide a common metric of alcohol exposure, regardless of drink type (e.g., beer, wine, liquor). Using standard drink equivalents, the QFI denotes the average ounces of absolute alcohol consumed per day. A QFI of 0.6 reflects an average of ∼ 1 standard drink/day. For clarity, QFI was converted to the estimated average number of drinks/day (D/D).

### Laboratory assessment

Prior to testing, participants underwent analysis for breath alcohol concentration (BrAC) and completed a urine screen to ensure the absence of disallowed drugs. Subsequently, a trained research assistant confirmed health status and substance use patterns. Participants detailed alcohol use for the prior 30 days. Participants who endorsed current use of THC products were asked to report the typical frequency of use (estimated days/week) in the last month.

### WM assessment description

**FIGURE 1:**
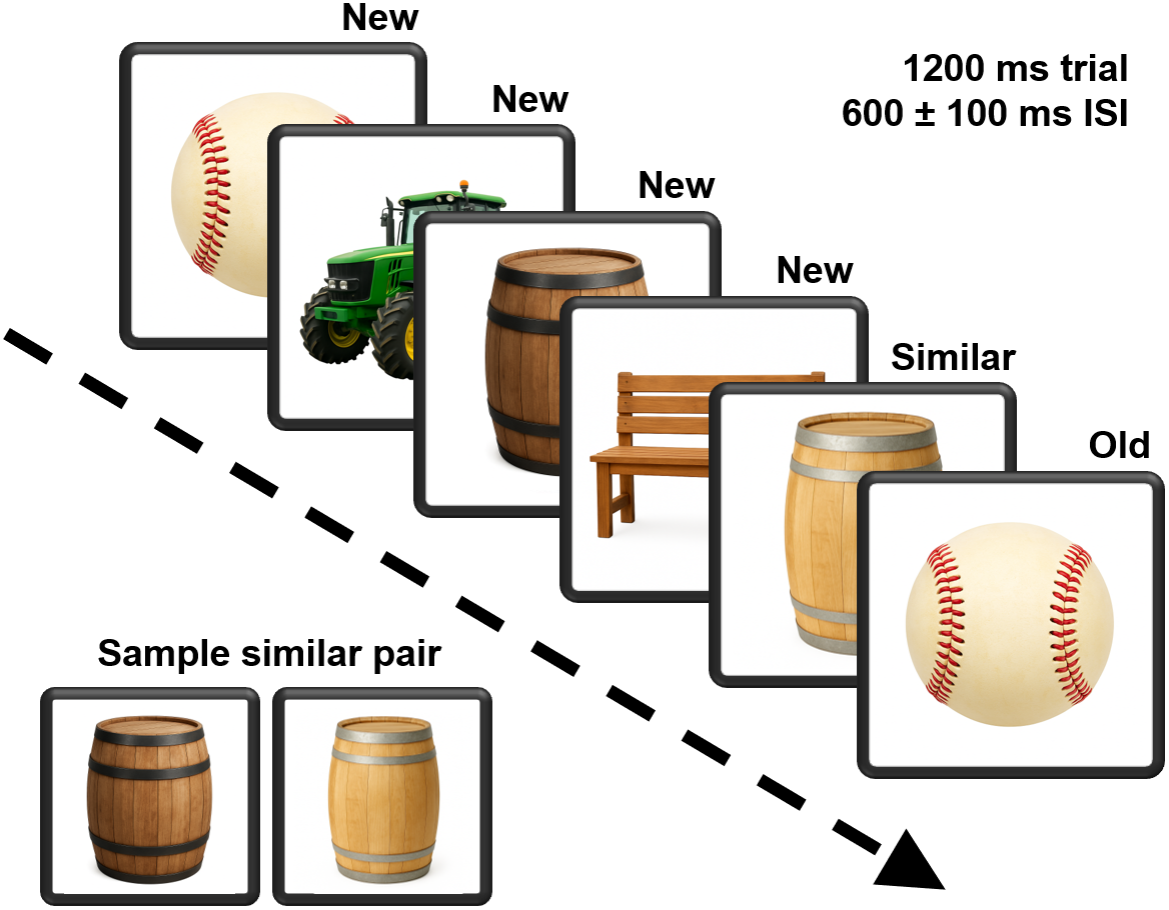
A depiction of the continuous recognition version of the Mnemonic Similarity Task. Pictures of neutral items were presented for 1200 ms, followed by a 600 ms interstimulus interval that varied by a ±100 ms jitter. Participants labeled each picture as either “new” or “old.” An example pairing of similar stimuli is also demonstrated. Task stimuli were obtained from the Stark Lab (www.github.com/celstark/MST). For publication purposes, images in the figure were created (GPT Image 1) to approximate those used in the task.

The Mnemonic Similarity Task (MST) (Stark et al., 2019) is well-validated in study of cognitive aging with demonstrated sensitivity to medial temporal lobe functions (Stark et al., 2015; Yassa et al., 2010). Unlike a typical n-back task, participants are not required to remember a specific interval (e.g., 2-back). Instead, as individual stimuli were displayed, participants judged each as either “old” if it had appeared in the list earlier or “new” if it had not. The task included 4 runs with each run composed of 124 trials including 40 identical pairs wherein 1 stimulus was presented a second time, 40 similar pairs wherein a second, similar stimulus was presented, and 44 stimuli were unique appearing only once with no similar stimuli shown. Stimulus order was pseudo-randomized with the constraint that the number of items between the initial presentation and the repeated presentation of an identical pair and that between the initial stimulus and its similar pair item varied by 2–8 items. Individuals were required to move the index finger of their dominant hand from a “home” position located between two response buttons housed in a response panel. Instructions were presented verbally while simultaneously displayed on the computer monitor. Practice trials were administered prior to initiating the task. Instructions emphasized the importance of performing as accurately and as quickly as possible. Figure 1 depicts the task. The complexity of the test permits a variety of approaches to parsing the stimuli. In this analysis the new stimulus condition included the unique stimuli and the first of a stimulus pair for which a second similar or identical stimulus was presented. The identical stimulus condition included only the 2nd stimulus in the “identical” pairing, and the similar condition, the 2nd stimulus in a “similar” pairing.

Testing occurred in a sound-attenuated, electrically shielded booth. Stimuli were presented on a monitor placed 109 cm in front of the participants. Stimuli subtended a visual angle of 8 × 8*^o^*. Individual stimuli were shown for 1200 ms during which participants made their response. Stimuli were separated by a 600 ms interstimulus interval (jittered by 100 ms). Neurophysiological responses were obtained using a 64-site montage (Electro-cap International, Eaton, OH), ground at mid-forehead, reference being linked earlobes, and maintaining impedance at or below 10 kOhms. Eye-blinks are monitored by electrodes above and below the left eye. Signals were sampled at 500 Hz with a 16-bit resolution and signals were band-passed filtered between.01 and 100 Hz. Further processing was completed off-line using EEGLAB (version 2024.2.1, Delorme and Makeig, 2004) and ERPLAB (version 12.01, Lopez-Calderon and Luck, 2014). First, a 1 Hz high pass finite impulse response (FIR) filter was applied, followed by a 50 Hz low pass FIR filter. Channels were then automatically rejected using the CLEAN_RAWDATA EEGLAB Plugin, when flatlined for greater than 5 s, when having more line noise relative to signal by greater than 4 standard deviations, and when correlation to neighboring channels was lower than 0.8. Data periods with significant noise were removed using CLEAN_RAWDATA when channel root mean square was greater than 7 standard deviations. Blink and gross muscle artifact components were automatically identified and removed from the EEG signal if the probability of being a blink or gross muscle artifact was greater than 0.9 based on independent component analysis (ICA) and the pop_icflag function within the EEGLAB toolkit. Flagged ICs were subtracted from the data. Embedded triggers permitted ERP analysis based on stimulus type and behavioral response.

### Analytic strategy

Behavioral and electrophysiological data were analyzed in separate parallel models. Depending on the presence of repeated measures, we fit either ordinary least squares regression models or linear mixed-effects models with random intercepts for participants. Correlations between typical alcohol use (D/D) and potential outcomes of interest were examined in preliminary analyses and used to guide model selection (see below). All models included age and education as nuisance covariates. Sex was excluded as a covariate because the average number of D/D was substantially higher (*M* = 1.8 vs. 1.1) and more dispersed (SD = 1.5 vs. 0.9) in men, creating unequal leverage and limited overlap. That said, recognizing their potential significance, we also analyzed, as a cautionary step, preliminary models including sex. These models failed to detect sex effects or sex-contingent moderation of drinking levels. Given the low proportion of users (∼22%), THC use was omitted from the primary model to improve estimation precision, however, *post hoc* sensitivity analyses were conducted to assess model stability in the THC-negative subsample. Exploratory models were constructed to probe the potential effects of THC use frequency on both behavioral and electrophysiological outcomes.

## Results

### Descriptive data/correlations

Demographic data, average D/D, and THC use information use are summarized in Table 1. Also shown are the summary statistics for the measures of depression (PHQ-9, Kroenke et al., 2001) and anxiety (GAD-7, Spitzer et al., 2006). These measures of negative affect were administered to account for its potential role as a covariate. Neither correlated with neurobiobehavioral measures or average D/D. Consequently, they were not considered further.

**TABLE 1:** Demographics.

Variables	Men (*N* = 54)	Women (*N* = 70)	Total (*N* = 124)
Age	70.30 (3.37) [65.00–76.00]	71.10 (4.36) [65.00–80.00]	70.75 (3.96) [65.00–80.00]
Education (years)	16.98 (2.48) [12.00–20.00]	17.36 (2.07) [13.00 - 20.00]	17.19 (2.26) [12.00 - 20.00]
Typical drinks per day	1.75 (1.51) [0.05–5.80]	1.04 (0.89) [0.02–3.31]	1.35 (1.25) [0.02–5.80]
**THC use**			
No	41 (75.9%)	56 (80.0%)	97 (78.2%)
Yes	13 (24.1%)	14 (20.0%)	27 (21.8%)
THC frequency (∼ days/week in prior month)	2.57 (1.34) [1.00–5.00]	3.11 (1.37) [1.00–5.00]	2.88 (1.36) [1.00–5.00]
Anxiety^1^	2.09 (2.23) [0.00–8.00]	3.96 (4.26) [0.00–20.00]	3.15 (3.63) [0.00–20.00]
Depression^2^	2.48 (2.52) [0.00–9.00]	3.41 (4.00) [0.00–19.00]	3.01 (3.46) [0.00–19.00]

^1^Anxiety measure GAD-7;

^2^Depression measure PHQ-9.

### Behavioral outcomes

**FIGURE 2:**
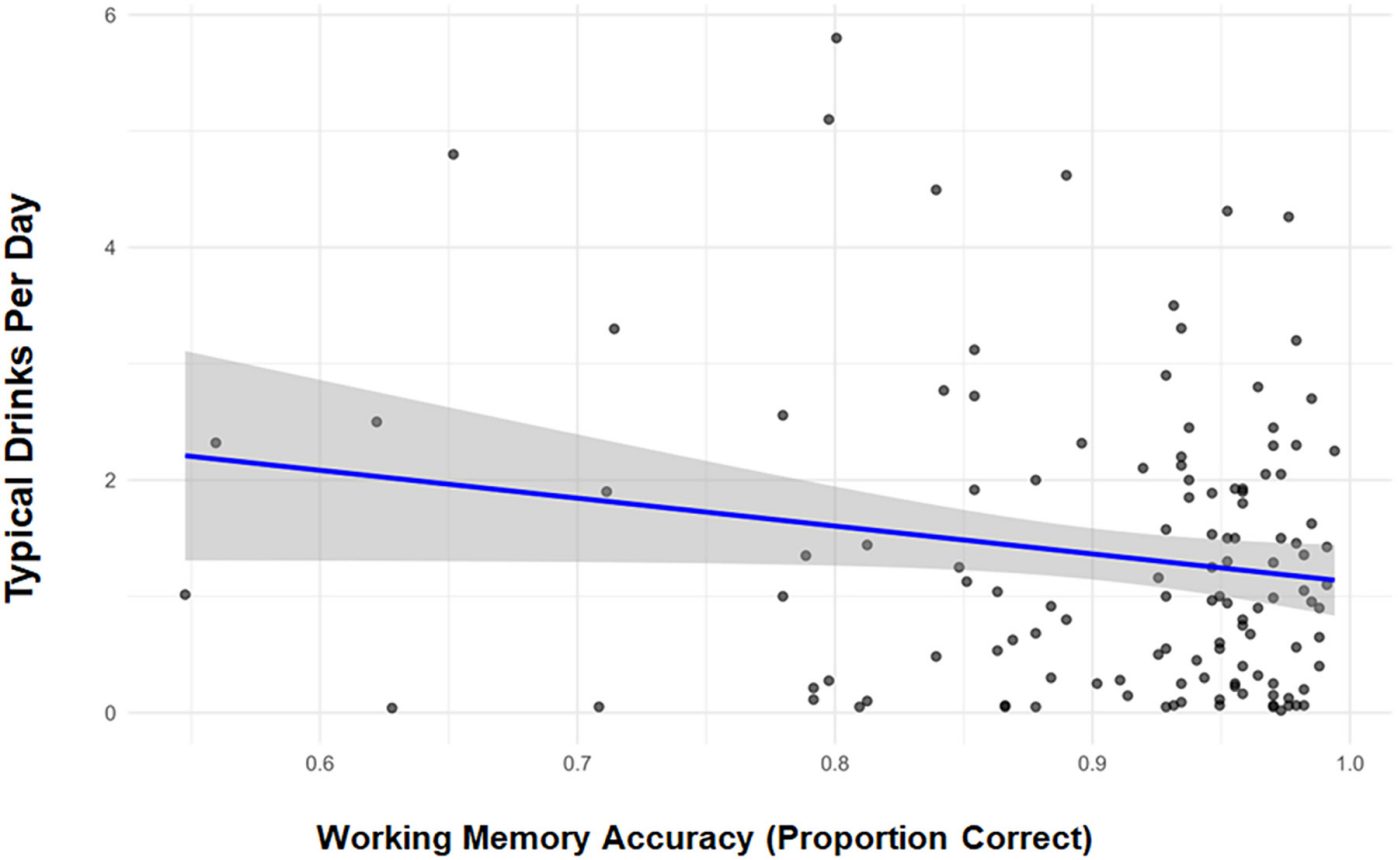
Correlation scatterplot depicting Mnemonic Similarity Task performance (mean proportion correct) to new stimuli and typical standard drinks per day.

Table 2 provides the raw mean accuracy and response times (accurate responses) by stimulus type. Correlations between D/D, accuracy, and response times were examined within each MST condition. The only notable association was between D/D and accuracy for “new” stimuli (*r* = −0.17, *p* = 0.053), thus the behavioral model was confined to this condition. The model revealed reductions in accuracy with increasing D/D (β = −0.013, 95% CI [−0.026, −0.001]; *p* = 0.040). This relationship is depicted in Figure 2.

**TABLE 2:** Behavioral performance by stimulus condition.

Condition	Mean% correct	SD%	Min%	Max%	Mean RT (secs)	SD	Min	Max
Identical	66%	19%	10%	96%	0.93	0.07	0.71	1.08
New	91%	9%	55%	99%	0.78	0.07	0.6	0.95
Similar	28%	12%	6%	61%	0.85	0.08	0.64	1.06

Despite null effects observed in the development of the full model, in *post hoc*/exploratory analyses we ask whether, when the sexes were interrogated separately, the association between D/D and performance differed. The resulting analyses suggested significant QFI effects among men, but not women (β = −0.019 vs. β = −0.009, respectively). Importantly, the constrained range in women’s D/D likely limited the opportunity to observe a relationship. Sensitivity analyses, wherein the THC users were excluded, indicated a similar alcohol effect (β = −0.018, 95% CI [−0.032, −0.004]; *p* = 0.014). Constrained by sample size (*n* = 27), exploratory analyses of THC users detected no effect of THC use frequency (β = −0.028, 95% CI [−0.060, 0.005], *p* = 0.091). *Post hoc* tests comparing persons with no history of smoking to those with any history of smoking on performance in the alcohol-sensitive new stimulus condition did not achieve significance [t (1,118) = 1.73; *p* = 0.09)].

### Neurophysiological outcomes

To explore neurophysiological correlates, mean amplitudes for the anterior cluster (Fz, F3, F4) and a posterior/parietal cluster (Pz, P3, P4) were derived for each stimulus type. From the anterior/mid-frontal sites, we derived the mean amplitude of the negative going shift occurring between 300 and 500 ms (i.e., FN400) after stimulus presentation. Within the posterior/parietal cluster, we examined the positive-going shift occurring between 550 and 800 ms after stimulus presentation (i.e., LPC). As noted in Table 2, the accuracy rates were highly disparate across the stimulus conditions, creating significant concerns regarding the precision and reliability assessing relationships between accuracy as a main or interaction effect in analysis of the ERP components. Therefore, accuracy was not included in the statistical models. The primary models included D/D and stimulus condition with age and education as nuisance covariates. The accepted number of trials for each of the components by stimulus type are: similar stimuli (*N* = 80), the median = 76, interquartile range 71–79, range 39–80; for identical (*N* = 80), median = 77, interquartile range 72–79, range 34–80; for new, (*N* = 336) median = 321, interquartile range 305–329, range 211–336.

The primary FN400 model did not detect a D/D effect (β = −0.167, 95% CI [−0.449, 0.115], *p* = 0.246), or any effects of condition (*p* > 0.107). Exploratory analyses detected no effect of THC use frequency on FN400 mean amplitude (β = −0.480, 95% CI [−1.442, 0.483], *p* = 0.326).

The primary LPC model (see Table 3) revealed a D/D effect with increased alcohol consumption being associated with reduced LPC amplitudes (β = −0.214, 95% CI [−0.412, −0.016], *p* = 0.034). A condition effect was also observed; mean amplitude was lower in the “new” condition relative to both “identical” (β = −0.680, 95% CI [−0.942, −0.418], *p* < 0.001) and “similar” conditions (β = −0.490, 95% CI [−0.228, −0.751], *p* < 0.001). Sensitivity analyses removing THC users reduced the D/D effect to a statistical trend, yet, critically, retained the magnitude and directionality of the D/D effect (β = −0.205, 95% CI [−0.448, 0.037], *p* = 0.097). Guided by the behavioral data, a *post hoc* model examining LPC amplitude in “new” trials was conducted and detected a D/D effect consistent with the full model (β = −0.229, 95% CI [−0.406, −0.053], *p* = 0.011). Again, outcomes suggested stronger QFI effects among men (β = −0.210 vs. β = −0.009, respectively) As anticipated from the behavioral and FN400 analyses, exploratory analyses detected no effect of THC use frequency (β = −0.130, 95% CI [−0.227, 0.487], *p* = 0.474). Finally, to examine consistency, the correlation between LPC mean amplitude in response to “new” stimuli and behavioral performance in the same stimulus condition was examined and indicated a significant relationship (*r* = 0.301, *p* = 0.001). As in the behavioral data, the test of LPC mean amplitude differences between and “never” and “ever” smokers revealed no significant difference [t (1,116) = 1.44, *p* = 0.15)].

**TABLE 3:** Late positive component full model.

Predictors	Estimates	CI	p
Typical drinks per day	−0.214	−0.412 to −0.016	**0.034**
Age	−0.035	−0.097 to 0.028	0.274
Education (years)	0.045	−0.065 to 0.155	0.422
Condition [New]	−1.190	−1.542 to −0.838	**<0.001**
Condition [Similar]	−1.195	−1.547 to −0.843	**<0.001**
**N**	**119**		
Observations	713		
Marginal R^2^/conditional R^2^	0.092/0.494		

The referent for condition is the “Identical Stimuli.” Five individuals were excluded from EEG analysis due to insufficient trials within specific conditions and/or were not able to complete EEG testing due to scheduling conflicts or technical difficulties. Bold font depicts *p* values of < 0.05.

**FIGURE 3:**
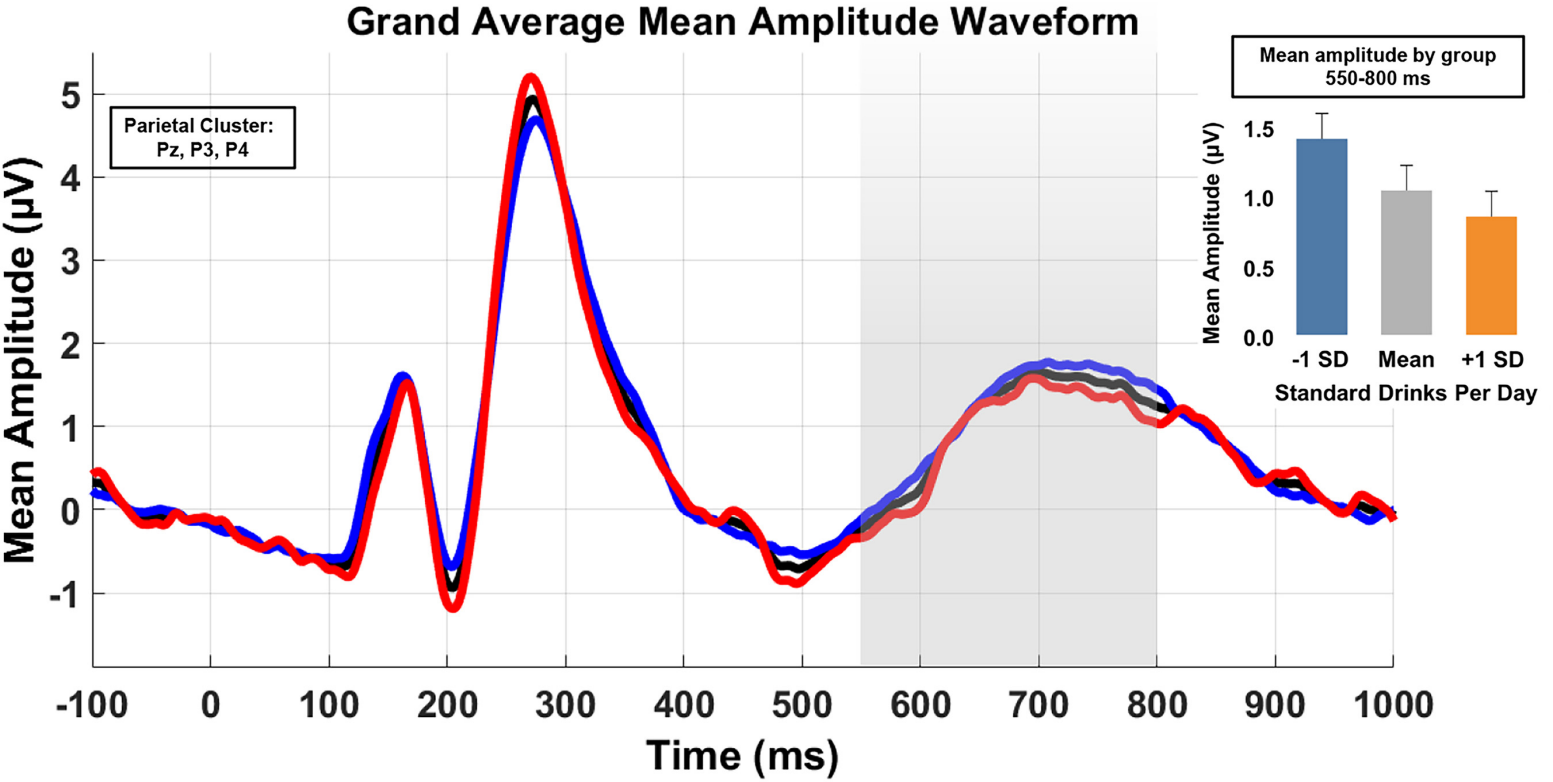
Grand average waveforms of mean amplitude for the parietal cluster (electrodes Pz, P3, and P4) across the 550–800 ms post stimulus onset. Analyses treated D/D as a continuous variable. However, for illustration purposes, low and high drinking groups were derived from a median split. The black line is the waveform for the full sample, the blue line is the low drinking group, and the high drinking group is in red. An inserted histogram depicts the predicted sample LPC mean amplitude and the predicted means for ± SD.

Figure 3 depicts the grand mean waveform for the parietal cluster. The epoch of interest (550–800 ms) is highlighted in gray. Demonstrating the effect of alcohol use when defined as a continuous variable was impracticable. Therefore, for the purposes of illustration only, drinkers were sub-grouped into “high” vs “low” using a median split. The black line depicts the full sample; the red and blue lines depict high and low drinkers, respectively. For additional clarification, the inserted histogram illustrates the predicted mean amplitude for the overall sample and that for groups differentiated by ± 1 SD.

## Discussion

Age-related cognitive decline presents a significant threat to the health and well-being of individuals, families and communities. With a larger proportion of older adults (> 65) continuing to consume alcohol and compelling data demonstrating alcohol’s negative consequences on neurobiobehavioral processes, there are increasing concerns that its use may exacerbate normal aging processes, even among older drinkers without histories of AUD. Despite increasing awareness and attention in the media, programmatic scientific study lags. The current work using data obtained in the course of baseline assessment in a longitudinal study of healthy community-residing older drinkers, represents an initial step to mitigate this gap.

Overall, the outcomes suggest that, even in the absence of chronic excessive use, alcohol drinking may be inversely related to WM performance and accompanied by differential shifts in underlying neural dynamics. Unsurprisingly, in light of well-documented heterogeneity, alcohol effects were modest and were not uniformly observed. We did not have *a priori* hypotheses regarding differential sensitivity as a function of either stimulus condition or anterior vs posterior ERPs. Thus, the findings indicating differences in the relationship between drinking level and recognition memory as a function of stimulus demands (identifying a stimulus as “new”) and between the two ERP components provide direction for future study. For example, the observed differences in alcohol effects on the ERP components suggest that the recollection component of recognition memory (reflected in the LPC) may be more vulnerable than is the familiarity component (reflected in the FN400) (Rugg and Curran, 2007; Yang et al., 2019). This contrast merits exploration. Interestingly, the vulnerability of neurobehavioral responses to “new” stimuli aligns with findings from previous work using an acute administration paradigm (Lewis et al., 2019), reinforcing study of alcohol’s effects on effective processing of novel stimuli.

Our findings are particularly striking in the context of the study design and selection criteria, which controlled for many individual variables known to confound interpretation of both alcohol and aging effects. We note that based on drinking levels and respondent characteristics in epidemiological reports, we might have expected null results or perhaps alcohol-related benefits. Adding another potential challenge was the relatively high rate of THC use in the sample and the resulting potential impact of its use on neurocognition. Thus, evincing negative alcohol effects under these conditions emphasizes the potential vulnerability of key neurobiobehavioral processes and functions and reinforces the need for comprehensive multi-method/multimodal study. Together, as we have proposed in other settings, the patterns motivate study of the interaction of alcohol sensitivity and differential risk for alcohol-related decline.

The significance of the study is also reflected in specific characteristics of the sample. For example, while women are underrepresented across alcohol studies, over half of the participants were women. Consistent with a large extant literature, typical drinking was higher in men than women. The absence of sex effects in preliminary analyses is noteworthy and consistent with other reports regarding sex differences in vulnerability to alcohol-related impairment. We note that the results from the *post hoc* analyses by sex, conducted in the absence of a sex main or interaction effect in the initial analysis wherein D/D was significantly related to performance among men, but absent in women, was unexpected. However, we urge strong caution in their interpretation. As noted in the analytic strategy, both the typical D/D and, notably, the range of D/D was lower in women than men. Thus, the ostensible sex differences may result from insufficient power to detect such an effect and/or it may be unrelated to sex, *per se*, driven instead by group differences in drinking levels. While the sample composition was inadequate for additional interrogation, the exploration of sex differences is a continuing priority.

The age range of the sample is a considerable strength. In earlier work, we encountered challenges in recruiting persons over age 70 (e.g., Gilbertson et al., 2009). Perhaps reflecting national trends, in this study, we recruited a significantly larger age range (65–80) with 10% of the sample entering the study at age 75 or above. This extended range enhances the generalization of findings and offers a significant advantage in clarifying age and alcohol interactions into older ages, as permitted via their longitudinal study.

As detailed previously, our goal was to study “typical” older drinkers. Thus, none of the participants had histories an SUD (excluding nicotine as described earlier), none reported problems associated with their use, and none reported the intention to modify their drinking over the next year. To permit focus on on-going drinking patterns and avoid inclusion of rare or occasional drinkers, minimal thresholds lying within current NIAAA guidelines were applied. To explore the range of drinking levels in healthy community drinkers, upper limits were not defined. As reflected in summary tables, drinking levels varied widely with a substantial number of drinkers exceeding current guidelines. A similar pattern was observed in an earlier independent sample of older community-residing drinkers (Lewis et al., 2018). Although provocative, the current sample distribution was insufficient to meaningfully interrogate the issue. That said, the data clearly indicate that many currently healthy older adults are not aware of potential threats and/or are not compelled to modify their drinking patterns.

## Conclusion

Together, the findings, albethey modest, reveal an age-related vulnerability to alcohol’s chronic effects, even in the absence of AUD. In this analysis of baseline data, age X alcohol vulnerability was interrogated in WM; more specifically recognition memory wherein both behavioral and neurophysiological outcomes obtained in the processing of new stimuli were negatively related to D/D. Additionally, while THC use did not account for a significant portion of the variance, the reported rates of THC use reinforce efforts to systematically investigate the comorbid use of THC and alcohol among older adults. One might seek to exclude current THC users from study. That said, such exclusion could result in less representative samples, particularly in light of the increasing use of THC in this age group. In summarizing these analyses, we note that being derived from a single assessment, they do not speak to alcohol’s potential effects on cognitive decline. These issues will be queried in analyses of the on-going longitudinal assessments.

## Limitations

One variable that may have limited the opportunity to observe more widespread alcohol effects is the sample’s relatively high educational level. While generally reflecting the surrounding aging community, it is higher than national statistics and suggests the need for expanding the catchment area in future work. Notably, despite the educational level, alcohol-related compromise was observed. By deriving the typical number of standard drinks/day, we met our intent to describe average typical drinking (D/D) in this sample of healthy older adults. We concede, however, that the method does not lend itself to analysis of specific drinking patterns (e.g., accounting for maximum drinks/chronic binge drinking). Despite this limitation, the data speak to substantive concerns regarding older adults. It also directs design of future work. Additionally, ideally, a comprehensive assessment probing diverse neuropsychological domains would be used. The parameters of this award were insufficient to support a more comprehensive protocol. Therefore, we leveraged understanding of cognitive aging and alcohol in task selection. Given our pilot work and prior reports using similarly aged participants, the poor performance in the similar stimulus condition poses an unexpected limitation which will be examined in interrogation of performance stability across the repeated assessments.

## Data Availability

The datasets presented in this article are not readily available because the project is still in data collection phase. Requests to access the datasets should be directed to SJN, sjnixon@ufl.edu.

## References

[B1] Aichele S. Rabbitt P. Ghisletta P. (2016). Think fast, feel fine, live long: A 29-year study of cognition, health, and survival in middle-aged and older adults. *Psychol. Sci.* 27 518–529. 10.1177/0956797615626906 26917212

[B2] Battista J. T. Vidrascu E. Robertson M. M. Robinson D. L. Boettiger C. A. (2025). Greater alcohol intake predicts accelerated brain aging in humans, which mediates the relationship between alcohol intake and behavioral inflexibility. *Alcohol Clin. Exp. Res.* 49 564–572. 10.1111/acer.15534 39985485 PMC11928243

[B3] Brandt J. Spencer M. Folstein M. (1988). The telephone interview for cognitive status (TICS). *Neuropsychiatry Neuropsychol. Behav. Neurol.* 1 111–117. 10.1002/gps.2503 21229597 PMC3832189

[B4] Breslow R. A. Castle I. P. Chen C. M. Graubard B. I. (2017). Trends in alcohol consumption among older americans: National health interview surveys, 1997 to 2014. *Alcohol Clin. Exp. Res.* 41 976–986. 10.1111/acer.13365 28340502 PMC5439499

[B5] Cabeza R. Albert M. Belleville S. Craik F. I. M. Duarte A. Grady C. L. (2018). Maintenance, reserve and compensation: The cognitive neuroscience of healthy ageing. *Nat. Rev. Neurosci.* 19 701–710. 10.1038/s41583-018-0068-2 30305711 PMC6472256

[B6] Cahalan D. Cissin L. Crossley H. (1969). *American drinking practices: A national study of drinking behaviors and attitudes (Monograph No. 6).* New Brunswick, NJ: Rutgers Center of Alcohol Studies.

[B7] Chosy E. J. Edland S. Launer L. White L. R. (2022). Midlife alcohol consumption and later life cognitive impairment: Light drinking is not protective and APOE genotype does not change this relationship. *PLoS One* 17:e0264575. 10.1371/journal.pone.0264575 35275952 PMC8916616

[B8] Delorme A. Makeig S. (2004). EEGLAB: An open source toolbox for analysis of single-trial EEG dynamics including independent component analysis. *J. Neurosci. Methods* 134 9–21. 10.1016/j.jneumeth.2003.10.009 15102499

[B9] Funk-White M. Wing D. Eyler L. T. Moore A. A. Reas E. T. McEvoy L. (2023). Neuroimaging-derived predicted brain age and alcohol use among community-dwelling older adults. *Am. J. Geriatr. Psychiatry* 31 669–678. 10.1016/j.jagp.2023.02.043 36925380 PMC11534213

[B10] Gilbertson R. Ceballos N. A. Prather R. Nixon S. J. (2009). Effects of acute alcohol consumption in older and younger adults: Perceived impairment versus psychomotor performance. *J. Stud. Alcohol. Drugs* 70 242–252. 10.15288/jsad.2009.70.242 19261236 PMC2653610

[B11] Han B. H. Palamar J. J. (2020). Trends in cannabis use among older adults in the United States, 2015-2018. *JAMA Intern. Med.* 180 609–611. 10.1001/jamainternmed.2019.7517 32091531 PMC7042817

[B12] Hassing L. B. (2018). Light alcohol consumption does not protect cognitive function: A longitudinal prospective study. *Front. Aging Neurosci.* 10:81. 10.3389/fnagi.2018.00081 29632484 PMC5879951

[B13] Kepner W. E. Han B. H. Nguyen D. Han S. S. Lopez F. A. Palamar J. J. (2023). Past-month binge drinking and cannabis use among middle-aged and older adults in the United States, 2015-2019. *Alcohol* 107 32–37. 10.1016/j.alcohol.2022.07.006 35934163 PMC9933134

[B14] Keyes K. M. (2023). Alcohol use in the older adult US population: Trends, causes, and consequences. *Alcohol* 107 28–31. 10.1016/j.alcohol.2022.05.005 35661693

[B15] Kroenke K. Spitzer R. L. Williams J. B. (2001). The PHQ-9: Validity of a brief depression severity measure. *J. Gen. Intern. Med.* 16 606–613. 10.1046/j.1525-1497.2001.016009606.x 11556941 PMC1495268

[B16] Kwon S. Rugg M. D. Wiegand R. Curran T. Morcom A. M. (2023). A meta-analysis of event-related potential correlates of recognition. *Psychon. Bull. Rev.* 30 2083–2105. 10.3758/s13423-023-02309-y 37434046 PMC10728276

[B17] Lewis B. Garcia C. C. Nixon S. J. (2018). Drinking patterns and adherence to “low-risk” guidelines among community-residing older adults. *Drug Alcohol Depend.* 187 285–291. 10.1016/j.drugalcdep.2018.02.031 29698895 PMC6324529

[B18] Lewis B. Garcia C. C. Boissoneault J. Price J. L. Nixon S. J. (2019). Working memory performance following acute alcohol: Replication and extension of dose by age interactions. *J. Stud Alcohol Drugs* 80 86–95. 10.15288/jsad.2019.80.86 30807279 PMC6396508

[B19] Lobo E. Dufouil C. Marcos G. Quetglas B. Saz P. Guallar E. (2010). Is there an association between low-to-moderate alcohol consumption and risk of cognitive decline? *Am. J. Epidemiol.* 172 708–716. 10.1093/aje/kwq187 20699263

[B20] Lopez-Calderon J. Luck S. J. (2014). ERPLAB: An open-source toolbox for the analysis of event-related potentials. *Front. Hum. Neurosci.* 8:213. 10.3389/fnhum.2014.00213 24782741 PMC3995046

[B21] Moussa M. N. Simpson S. L. Mayhugh R. E. Grata M. E. Burdette J. H. Porrino L. J. (2014). Long-term moderate alcohol consumption does not exacerbate age-related cognitive decline in healthy, community-dwelling older adults. *Front. Aging Neurosci.* 6:341. 10.3389/fnagi.2014.00341 25601835 PMC4283638

[B22] Nasreddine Z. S. Phillips N. A. Bedirian V. Charbonneau S. Whitehead V. Collin I. (2005). The montreal cognitive assessment, MoCA: A brief screening tool for mild cognitive impairment. *J. Am. Geriatr. Soc.* 53 695–699. 10.1111/j.1532-5415.2005.53221.x 15817019

[B23] Nixon S. J. Lewis B. (2020). Brain structure and function in recovery. *Alcohol. Res.* 40:4. 10.35946/arcr.v40.3.04 33282611 PMC7703868

[B24] Nixon S. J. Garcia C. C. Lewis B. (2023). Age as a potential modulator of alcohol-related deficits. *Alcohol* 107 12–18. 10.1016/j.alcohol.2022.07.004 35940507

[B25] Oscar-Berman M. Marinkovic K. (2007). Alcohol: Effects on neurobehavioral functions and the brain. *Neuropsychol. Rev.* 17 239–257. 10.1007/s11065-007-9038-6 17874302 PMC4040959

[B26] Reas E. T. Laughlin G. A. Kritz-Silverstein D. Barrett-Connor E. McEvoy L. K. (2016). Moderate, regular alcohol consumption is associated with higher cognitive function in older community-dwelling adults. *J. Prev. Alzheimers Dis.* 3 105–113. 10.14283/jpad.2016.89 27184039 PMC4866612

[B27] Rugg M. D. Curran T. (2007). Event-related potentials and recognition memory. *Trends Cogn. Sci.* 11 251–257. 10.1016/j.tics.2007.04.004 17481940

[B28] Sabia S. Elbaz A. Rouveau N. Brunner E. J. Kivimaki M. Singh-Manoux A. (2014). Cumulative associations between midlife health behaviors and physical functioning in early old age: A 17-year prospective cohort study. *J. Am. Geriatr. Soc.* 62 1860–1868. 10.1111/jgs.13071 25283337 PMC4206608

[B29] Salthouse T. (2012). Consequences of age-related cognitive declines. *Annu. Rev. Psychol.* 63 201–226. 10.1146/annurev-psych-120710-100328 21740223 PMC3632788

[B30] Sebastian A. Baldermann C. Feige B. Katzev M. Scheller E. Hellwig B. (2013). Differential effects of age on subcomponents of response inhibition. *Neurobiol. Aging* 34 2183–2193. 10.1016/j.neurobiolaging.2013.03.013 23591131

[B31] Spitzer R. L. Kroenke K. Williams J. B. Lowe B. (2006). A brief measure for assessing generalized anxiety disorder: The GAD-7. *Arch. Intern. Med.* 166 1092–1097. 10.1001/archinte.166.10.1092 16717171

[B32] Spreng R. N. Turner G. R. (2019). The shifting architecture of cognition and brain function in older adulthood. *Perspect. Psychol. Sci.* 14 523–542. 10.1177/1745691619827511 31013206

[B33] Stark S. M. Kirwan C. B. Stark C. E. L. (2019). Mnemonic similarity task: A tool for assessing hippocampal integrity. *Trends Cogn. Sci.* 23 938–951. 10.1016/j.tics.2019.08.003 31597601 PMC6991464

[B34] Stark S. M. Stevenson R. Wu C. Rutledge S. Stark C. E. (2015). Stability of age-related deficits in the mnemonic similarity task across task variations. *Behav. Neurosci.* 129 257–268. 10.1037/bne0000055 26030427 PMC4451612

[B35] Sullivan E. V. Pfefferbaum A. (2019). Brain-behavior relations and effects of aging and common comorbidities in alcohol use disorder: A review. *Neuropsychology* 33 760–780. 10.1037/neu0000557 31448945 PMC7461729

[B36] Topiwala A. Allan C. L. Valkanova V. Zsoldos E. Filippini N. Sexton C. (2017). Moderate alcohol consumption as risk factor for adverse brain outcomes and cognitive decline: Longitudinal cohort study. *BMJ* 357:j2353. 10.1136/bmj.j2353 28588063 PMC5460586

[B37] Yang H. Laforge G. Stojanoski B. Nichols E. S. McRae K. Kohler S. (2019). Late positive complex in event-related potentials tracks memory signals when they are decision relevant. *Sci. Rep.* 9:9469. 10.1038/s41598-019-45880-y 31263156 PMC6603184

[B38] Yang Y. Wang D. Hou W. Li H. (2023). Cognitive decline associated with aging. *Adv. Exp. Med. Biol.* 1419 25–46. 10.1007/978-981-99-1627-6_3 37418204

[B39] Yassa M. A. Stark S. M. Bakker A. Albert M. S. Gallagher M. Stark C. E. (2010). High-resolution structural and functional MRI of hippocampal CA3 and dentate gyrus in patients with amnestic Mild cognitive impairment. *Neuroimage* 51 1242–1252. 10.1016/j.neuroimage.2010.03.040 20338246 PMC2909476

[B40] Zahr N. M. (2024). Alcohol use disorder and dementia: A review. *Alcohol. Res.* 44:3. 10.35946/arcr.v44.1.03 38812709 PMC11135165

